# Percutaneous ethanol and calcitriol injection therapy for hyperparathyroidism – a single-centre experience

**DOI:** 10.3389/fendo.2025.1562493

**Published:** 2025-06-04

**Authors:** Eugene Kwong Fei Leong, Ray Meng See, Zhimin Lin, Meredeth Choon Siang Chin, Joshua Wei Yang Chew, Kee Yuan Ngiam, James Wai Kit Lee

**Affiliations:** ^1^ Division of General Surgery (Endocrine & Thyroid Surgery), Department of Surgery, National University Hospital, Singapore, Singapore; ^2^ Yong Loo Lin School of Medicine, National University of Singapore, Singapore, Singapore; ^3^ Breast Surgery, Emergency & General Surgery, Khoo Teck Puat Hospital, Singapore, Singapore; ^4^ Department of Surgery, Alexandra Hospital, Singapore, Singapore

**Keywords:** hyperparathyroidism, parathyroid adenoma, percutaneous injection, case series, calcium

## Abstract

**Background:**

The purpose of this study is to evaluate the use of percutaneous ethanol and calcitriol injection therapy for hyperparathyroidism (HPT), while taking into account the efficacy, safety and feasibility as an ambulatory procedure alternative to surgical parathyroidectomy.

**Methods:**

We included nine patients who underwent percutaneous injection therapy for HPT from January 2018 to December 2021 in our institution. They were followed up from date of first percutaneous injection until death or October 2022 (mean duration of 9.0 months).

**Results:**

Four patients underwent percutaneous ethanol injection therapy (PEIT) (mean age 61.0 [31–89] years old), while the remaining five underwent percutaneous calcitriol therapy (PCIT) (mean age 62.6 [35–91] years old). The analyzed parameters are age, BMI, serum turn over markers as iPTH, Ca, alkaline phosphatase and vitamin D. Two out of the four patients undergoing PEIT had a successful outcome, although one needed to continue cinacalcet due to persistent serum calcium levels. Three out of five PCIT patients in our series had successful procedure, although one subsequently developed refractory disease.

**Conclusion:**

PEIT and PCIT are feasible and safe therapeutic alternatives to surgical parathyroidectomy in HPT refractory to medical treatment, with postulated benefits of decreased costs and being an outpatient procedure. However, further studies are necessary to evaluate the efficacy and cost-effectiveness with these techniques prior to widespread adoption.

## Introduction

The diagnosis of hyperparathyroidism (HPT) has increased with recognition that it is the most common cause of hypercalcaemia in the outpatient setting, and its association with chronic kidney disease (CKD) (1–4). Patients with secondary HPT (SHPT) are defined as having low or normal serum calcium levels initially, which induces the parathyroid glands to increase secretion of parathyroid hormone. On the other hand, patients with tertiary HPT (THPT) are defined as having high serum calcium levels initially, and both subgroups of patients can possibly have a background of CKD. HPT patients are more likely than the general population to develop cardiovascular and bone disease (1–3, 5–7). Pharmacological therapy in primary HPT (PHPT) patients not suitable for surgery includes calcimimetics or bisphosphates, while renal patients with SHPT or THPT can be treated with calcimimetics, phosphate binders and vitamin D receptor activators (3–5, 7, 8).

While parathyroidectomy surgery remains the definitive treatment for symptomatic PHPT and medically refractory renal HPT, significant risks include that of general anaesthesia, recurrent laryngeal nerve (RLN) injury and treatment failure (3, 5, 7). There is utility and interest in less invasive treatment modalities, which include percutaneous ethanol injection therapy (PEIT) and percutaneous calcitriol injection therapy (PCIT) (5, 9–14). PEIT involves ultrasonography-guided direct injection of ethanol into the parathyroid glands, which results in cellular death predominantly by coagulation (12).

PCIT is a similar procedure involving direct injection of calcitriol instead, and was developed in Japan as an alternative to PEIT in attempt to avoid risk of injury to surrounding tissues from ethanol leaking out of the parathyroid glands. The main benefit of PCIT over PEIT is demonstrated safety in the few available studies with no report of RLN palsy, which remains one of the major risks of PEIT (9, 11–15). Direct injection of calcitriol or maxacalcitol into hyperplastic parathyroids of uraemic patients results in apoptosis of parathyroid cells, observed on DNA electrophoresis and terminal deoxynucleotidyl transferase-mediated dUTP nick end-labeling (TUNEL) (16). When calcitriol is injected directly into the parathyroid glands, it enters the cells and binds to the vitamin D receptor (VDR) in the nucleus of parathyroid cells. This VDR activation increases the expression of pro-apoptotic proteins like BAX (Bcl-2 associated X protein) and Caspase-3, which will initiate the mitochondrial apoptotic pathway, leading to cell death. It is also postulated to suppress PTH secretion by achieving supernormal levels of calcitriol within parathyroid tissue (12). In addition, parathyroid volume on ultrasonography was significantly reduced at 4 and 12 weeks post-therapy. The success of PCIT over conventional oral calcitriol administration is postulated to be due to delivery of a higher concentration with direct injection (16). This is substantiated by a recent retrospective study 2022 which concludes that PCIT treatment helps to reduce iPTH hormone production in patients with hyperparathyroidism (27).

Our present study aims to evaluate these modalities in the management of HPT with regards to efficacy, safety and feasibility as an ambulatory procedure alternative to surgical parathyroidectomy.

## Methods

A retrospective cohort study of nine patients with primary or renal HPT undergoing PEIT or PCIT at National University Hospital between January 2018 and December 2021 was performed. Each subject was followed-up from date of first percutaneous injection until death or October 2022. Patients were selected for percutaneous injection therapy in accordance with guidelines published by the Japanese Working Group of PEIT of the Parathyroid in 2003 (17). In summary, selection criteria included: (i) intact PTH (iPTH) of 42.4 pmol/L or more; (ii) osteitis fibrosa or high bone turnover, confirmed radiologically or by bone metabolism markers; (iii) ultrasonography demonstration of enlarged parathyroid glands ≥0.5cm^3^, or suspected nodular hyperplasia; (iv) resistance to medical treatment; and (v) informed consent. Resistance to medical treatment was defined as serum PTH level above target range despite administration of administration of vitamin D or its analogues, with abnormal serum calcium and/or serum phosphate levels.

PEIT and PCIT was performed in an outpatient clinic setting under high-resolution ultrasonographic guidance with a Mindray DC-80A diagnostic ultrasound system (Shenzhen, China) and Mindray L12-3E linear transducer (3.0-12.0MHz). The dosage of ethanol or calcitriol injected was individualised according to calculated gland volume from ultrasonographic measurements (using the formula π/6 × a × b × c, where a, b and c represent gland dimensions), with injected volume in millilitres (ml) per gland corresponding to calculated volume in cubic centimetres (cm^3^) (13). For PEIT, 100% dehydrated ethanol was used (Martindale Pharmaceuticals, United Kingdom). For PCIT, 1 μg/ml calcitriol for intravenous therapy was used (Sterimax, Canada). Under ultrasonographic guidance, each disease parathyroid gland was localised, and the needle was carefully advanced until the tip was positioned within the gland’s centre. In patients with multiple enlarged parathyroid glands, all anomalous glands were injected in the same treatment setting, even in the event of bilateral disease. All our enrolled patients with primary HPT had single gland disease on imaging, suggestive of a single adenoma as the aetiology for the primary HPT. This was injected with PEIT or PCIT. Similarly, all visualised glands were injected for patients with renal hyperparathyroidism, with PEIT/PCIT injection volume corresponding to the calculated volume of each gland (volume ranging from 0.3ml to 3.5ml for each visualised gland). The patient was monitored in a day procedure ward for an hour after percutaneous injection therapy for potential complications such as dysphonia, bleeding, haematoma, anaphylaxis or airway compromise. There was no post-procedure hoarseness or haematoma observed clinically for any of the enrolled patients, and they did not declare any subjective post-procedure symptoms. As such, there was no objective requirement for post-procedure endoscopic evaluation for our study. Efficacy was assessed at 1, 3, 6 and 12 months after initial injection therapy with measurement of serum calcium adjusted for albumin, iPTH and alkaline phosphatase (ALP). If these biochemical markers demonstrated suboptimal response, the procedure was repeated at an interval of two to eighteen weeks. The criteria for subsequent treatments were for patients who had a less than 30% reduction in serum PTH and failure of normalisation of serum calcium levels. The same parathyroid glands were re-treated, with similar protocols to the initial treatment process (injected volume was proportionate to the calculated gland volume). Concomitant liver diseases were excluded from history and biochemistry analysis. Hypercalcaemia from iatrogenic replacement was excluded in our study’s patients prior to diagnosis of suboptimal or no response. None of the enrolled patients underwent parathyroidectomy or renal transplantation prior to the study period as well. Successful ablation was defined as a reduction of iPTH by more than 30% as documented in previous studies (25, 26). The study was conducted as a retrospective audit of an existing service at our institution and was approved for exemption by our institution’s domain specific review board (2021/00338). Our above methodology was chosen as a retrospective review of our specific institutional experience for surgical alternatives prior to further studies in the future. The baseline demographics and details of injection treatments of our patients have been included in **Tables 1** and **2** for reference.

**Table 1:** Patient demographics and baseline biochemistries.

Demographic details	PEIT group	PCIT group
Number of patients	4	5
Mean age, years (range)	61.0 (31-89)	62.6 (35-91)
Gender	4 Male	4 Male 1 Female
Mean body mass index, kg/m²	22.1	23.5
Cause of hyperparathyroidism	2 Primary 1 Secondary 1 Tertiary	1 Primary 2 Secondary 2 Tertiary
Mean serum iPTH, pmol/L (normal range 1.6-7.2 pmol/L)	140.9	210.2
Mean serum calcium, mmol/L (normal range 2.10-2.55 mmol/L)	3.01	2.55
Mean serum ALP, IU/L (normal range 30-110 IU/L)	942.0	553.6
Mean serum vitamin D, ug/L (normal range ≥30 ug/L)	18.4	18.7

**Table 2:** Details of injection treatments.

De-identified patients	Treatment group	Number and location of glands visualised on US	Dose/injection information	Number of injection treatments
PEIT 1	Ethanol	2 (right inferior and left superior)	2.5ml into right inferior, 0.5ml into left superior	2
PEIT 2	Ethanol	1 (right inferior)	0.5ml into right inferior	2
PEIT 3	Ethanol	1 (left inferior)	3.5ml into left inferior	1
PEIT 4	Ethanol	1 (right inferior)	0.3ml into right inferior	1
PCIT 1	Calcitriol	3 (right superior, right inferior, left inferior)	1.5ml into right superior, 0.5ml into right inferior, 0.5ml into left inferior	3
PCIT 2	Calcitriol	2 (right inferior, left superior)	1ml into right inferior, 1ml into left superior	2
PCIT 3	Calcitriol	3 (right inferior, right midpole, right superior)	1.5ml into right inferior, 1ml into right midpole, 1ml into right superior	3
PCIT 4	Calcitriol	1 (right superior)	2ml into right superior	1
PCIT 5	Calcitriol	1 (right inferior)	1ml into right inferior	1

## Results

**Figure 1:**
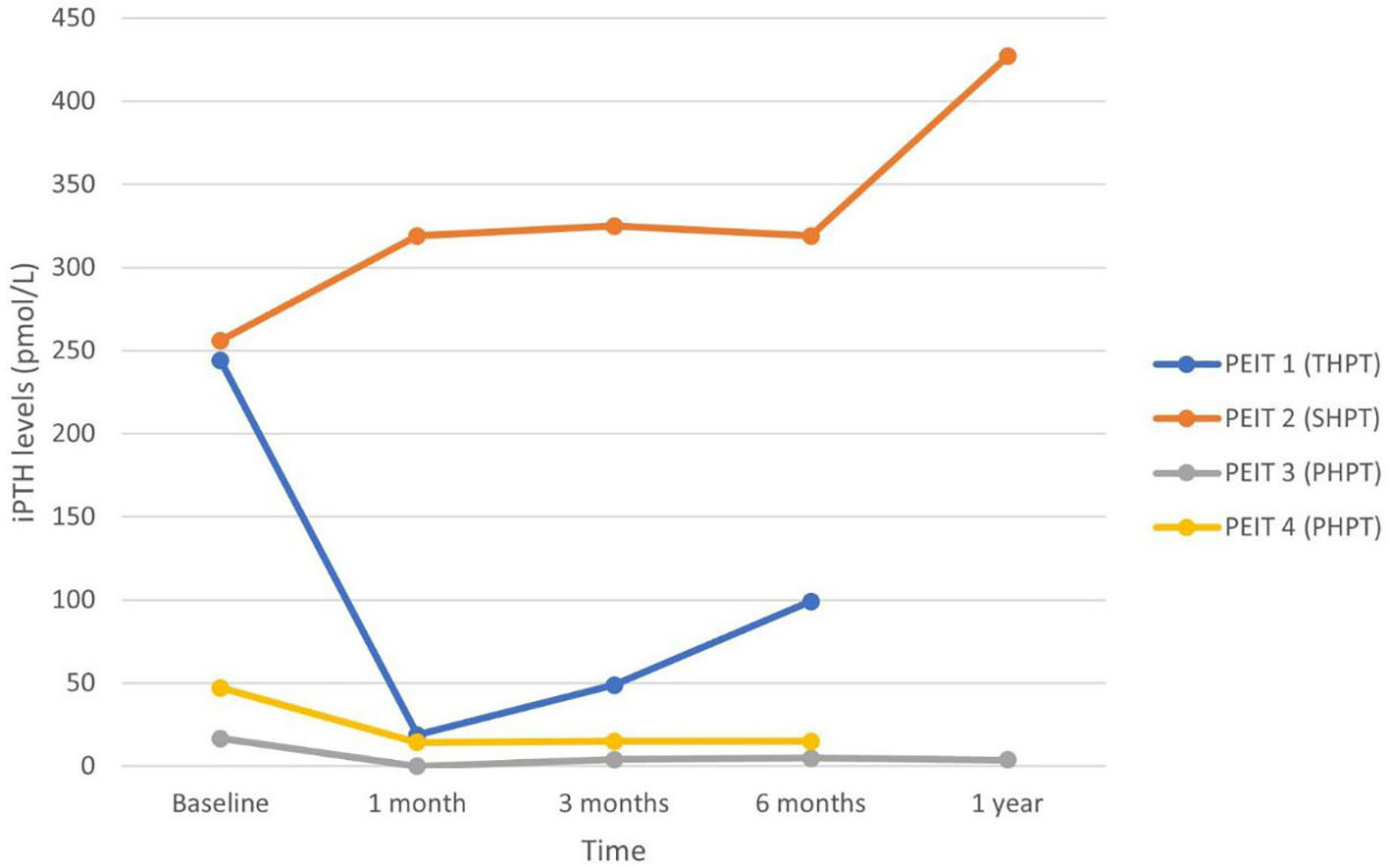
Serum iPTH in patients undergoing PEIT.

**Figure 2:**
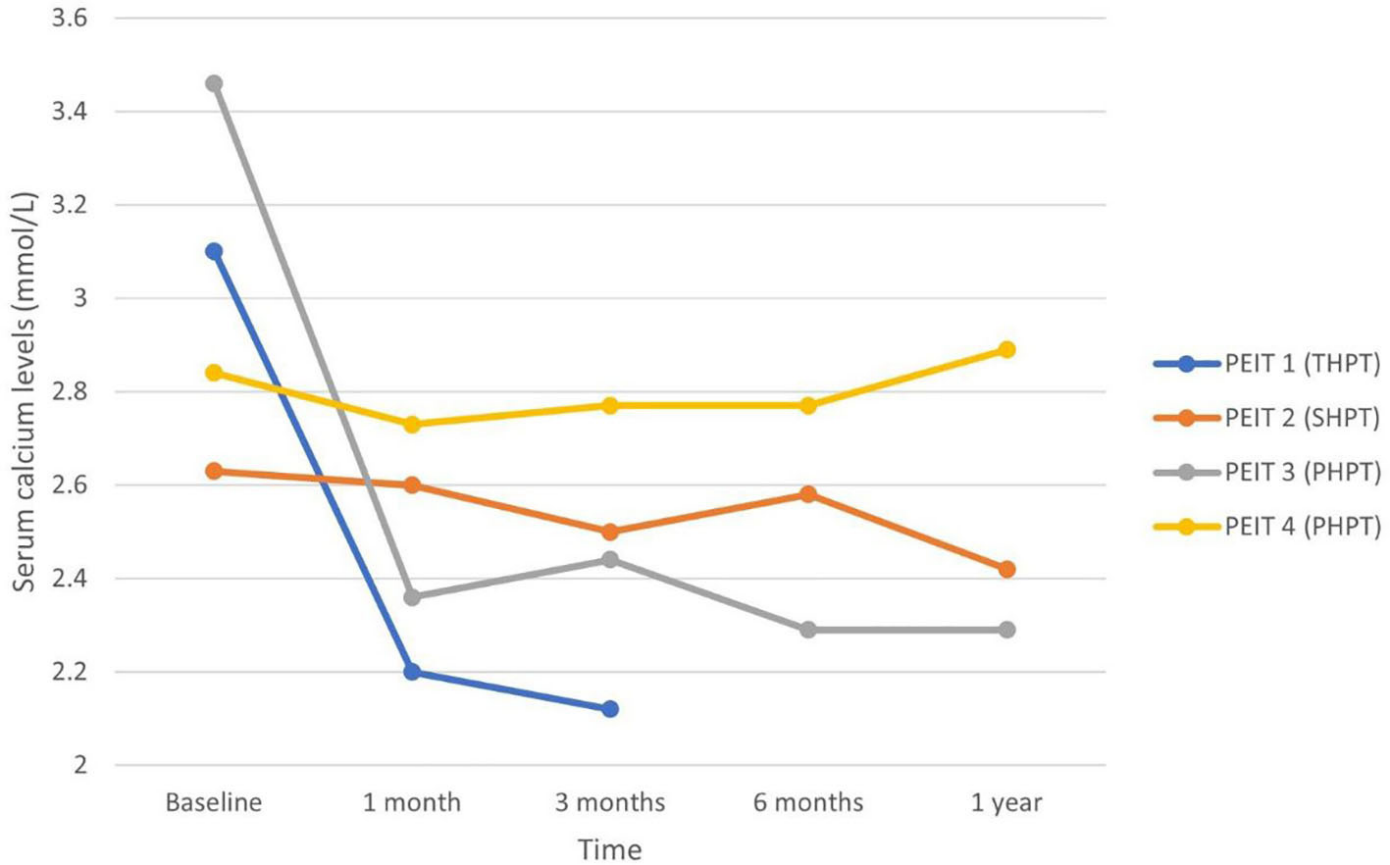
Serum calcium in patients undergoing PEIT.

**Figure 3:**
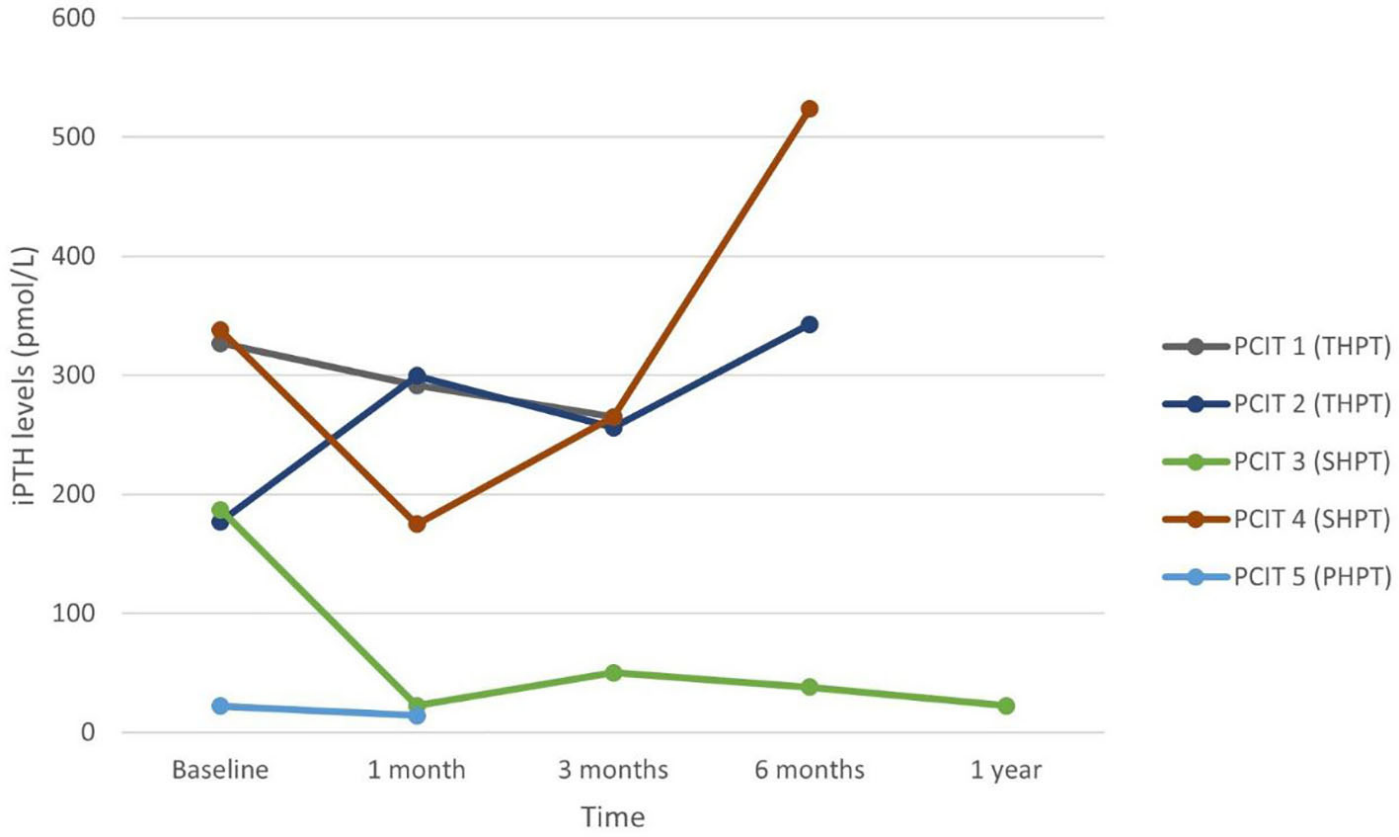
Serum iPTH in patients undergoing PCIT.

**Figure 4:**
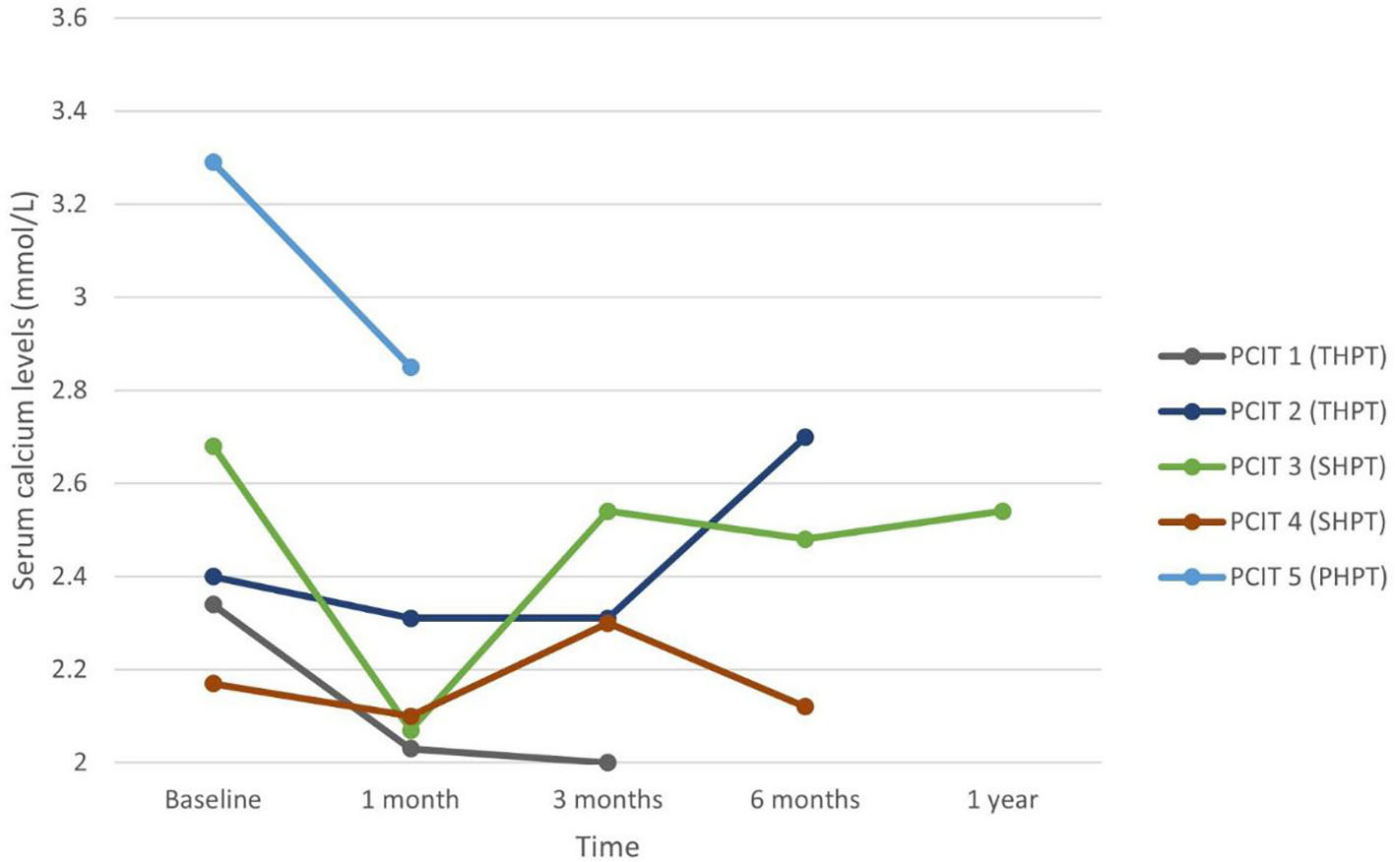
Serum calcium in patients undergoing PCIT.

Nine patients underwent percutaneous direction injection therapy for hyperparathyroidism between January 2018 and December 2021 in our institution – four with ethanol (PEIT) and five with calcitriol (PCIT). The mean age of patients undergoing PEIT was 61.0 years (range 31 to 89 years), and that of PCIT was 62.6 years (range 35 to 91 years). Mean duration of patient follow-up was 9.0 months. Each patient’s data was plotted and labelled with a different colour graph in **Figures 1**–**4**.

Two of the patients (PEIT 3 and 4) undergoing PEIT had PHPT from parathyroid adenoma, while Patient PEIT 2 had SHPT and Patient PEIT 1 had THPT – both from long-standing ESRF. All four patients were on 50 milligrams (mg) of cinacalcet daily. Patient PEIT 3 with PHPT underwent a single-course of PEIT and was successfully treated with normalisation of iPTH and adjusted calcium levels from 16.6 pmol/L and 3.46 mmol/L pre-procedure to 4.0 pmol/L and 2.44 mmol/L respectively at three months – cinacalcet was discontinued, and iPTH and adjusted calcium remained normal for the duration of study follow-up with no re-initiation of calcimimetic therapy or repeat PEIT. Patient PEIT 4 with PHPT had significant decrease in iPTH from 47.0 pmol/L to 14.3 pmol/L at one month, and 14.9 pmol/L at three months – iPTH remained at this level for the rest of the duration study. However, serum calcium level remained elevated in this patient, and he had to be continued on cinacalcet. Patient PEIT 1 who had THPT and ESRF on 21 years of haemodialysis experienced improvement in iPTH, adjusted calcium and ALP levels from 244.0 pmol/L, 3.1 mmol/L and 1667 IU/L pre-procedure to 18.8 pmol/L, 2.2 mmol/L and 306 IU/L respectively at one month, then to 48.8 pmol/L, 2.12 mmol/L and 162 IU/L respectively at three months after two courses of PEIT six weeks apart. Cinacalcet was held off for three months, but restarted afterward at original dose of 50mg daily when iPTH increased to 99.1 pmol/L six months of follow-up, despite a third course of PEIT one month prior. He died nine months after initial PEIT at the age of 59 years old, from reasons not related to hypercalcemia. PEIT was unsuccessful in the remaining patient PEIT 2 after one course of PEIT. He had SHPT from ESRF on peritoneal dialysis—baseline iPTH and ALP were 256 pmol/L and 2339 IU/L respectively.

Four patients undergoing PCIT had long-standing ESRF – two with SHPT (PCIT 3 and 4) and two with THPT (PCIT 1 and 2), while the last patient (PCIT 5) undergoing PCIT had PHPT from presumed parathyroid adenoma. All five patients were on daily cinacalcet dose of 50 mg. Patient PCIT 3 with ESRF on 12 years of haemodialysis had significant decrease in iPTH and ALP levels from 187.0 pmol/L and 741 IU/L to 22.4 pmol/L and 223 IU/L respectively at one month, then to 50.4 pmol/L and 144 IU/L respectively at three months. Cinacalcet was initially held off for three months, then restarted at a lower dose of 25mg daily thereafter and continued until end of the follow-up period. This patient had 3 courses of PCIT, with the subsequent courses two weeks and four-and-a-half months after the first. Both biochemistries continued to be better than pre-procedure – at 12 months post-procedure, iPTH was 22.4 pmol/L and ALP was 159 IU/L on half of the daily dose of cinacalcet prior to PCIT. Patient PCIT 5 with PHPT undergoing PCIT was a 91-year-old male with a 1.7-centimetre parathyroid adenoma and symptomatic hypercalcaemia refractory to medical treatment. He had significant comorbidities including moderate dementia, ischaemic heart disease and chronic kidney disease precluding surgery. He experienced improvement in iPTH and adjusted calcium levels from 22.2 pmol/L and 3.29 mmol/L to 7.3 pmol/L and 2.64 mmol/L respectively after one week, then worsened to 14.3 pmol/L and 2.85 mmol/L after one month although these biochemistries were still better than at baseline. ALP levels improved from 134 IU/L at baseline to 75 IU/L at one month. Unfortunately, this elderly patient died 6 weeks after treatment initiation from pneumonia, before further courses of PCIT could be performed for him. While a single administration of PCIT was not successful in resolving the patient’s hyperparathyroidism, there was evident biochemical response at one week and one month, suggesting that further treatments may have been useful. Patients PCIT 1 and 4 with renal hyperparathyroidism experienced some transient improvement in iPTH levels at 1 month follow-up from 338 pmol/L to 175 pmol/L, and from 327 pmol/L to 291.6 pmol/L – however iPTH and ALP continued to uptrend thereafter despite a second course of PCIT. PCIT was unsuccessful in patient PCIT 2 despite two attempts – he had THPT on haemodialysis for 10 years, and baseline iPTH and ALP levels were 177 pmol/L and 180 IU/L respectively.

There were no peri-procedural complications or mortality in the nine patients, accounting for a total of 17 courses of percutaneous injection therapy. All patients were treated in the ambulatory outpatient clinic setting with no requirement for procedural sedation, anaesthesia or hospitalisation.

## Discussion

While parathyroidectomy for HPT remains the most definitive treatment for HPT from parathyroid adenomas or secondary aetiologies such as long-standing ESRF, surgical management is accompanied by its own set of challenges, complications, and risks. Amongst these include treatment failure or recurrence due to inability to correctly identify the causative gland in PHPT or failure to remove all diseased glands in SHPT or THPT, including the infrequent supernumerary parathyroids, injury to the RLN resulting in dysphonia and rarely airway compromise. In addition, surgical parathyroidectomy is routinely performed under general anaesthesia to secure and protect the airway. PEIT and PCIT are options available in patients with high peri-operative risk, including patients with long-standing ESRF where risk of major cardiovascular adverse events (MACE) from general anaesthesia is elevated due to atherosclerosis and vascular calcification (1–3, 5–7, 10, 18).

Percutaneous injection therapy serves as an alternative treatment approach to surgical removal of the parathyroid glands for HPT refractory to medical treatment. It is a minimally-invasive procedure which can be performed in the ambulatory outpatient clinic as confirmed in our series, without the need for procedural sedation or general anaesthesia and their associated risks. All nine patients in our series underwent treatment as an office procedure without need for hospitalisation—associated benefits would include reduced utilisation of healthcare such as nursing, inpatient hospital beds, and operating theatre resources. For cost comparison, the average surgeon fee for a Parathyroidectomy in our specific institution is about SGD $7200-13200, while the anaesthetist fee is about SGD $2000-2900. Hospitalisation fee charges range from SGD $50–300 daily, with an average of 3-day inpatient hospitalisation stay for post-op recovery. In comparison, the estimated procedural costs of each PEIT/PCIT treatment is roughly SGD $1500 in our institution. As such, PEIT and PCIT modalities are postulated to be cheaper than surgical therapy as the need for the aforementioned resources are minimised, if not eliminated. Percutaneous injection therapy is relatively cheaper at about US$ 16.38 per injection dose for PCIT and US$ 29.35 for PEIT. In comparison, the mean incremental cost of parathyroidectomy in renal HPT patients is estimated at US$ 25,314—a majority of which arises from inpatient facility payments for the surgery, averaging at US$ 24,758 (19). Zanocco et al. concluded in a 2006 cost-effective analysis that calcimimetic treatment is not cost effective—total cost per year for cinacalcet treatment was US$ 7,095, but only resulted in 0.008 quality adjusted life-year (QALY) at an additional lifetime cost of US$ 176,097 (20). Komaba et al. reported in 2012 an incremental lifetime cost of US$ 27,858 with the Markov model when cinacalcet was added to standard treatment for Japanese SHPT patients eligible for parathyroidectomy (21). Iannazzo et al. described similar findings in 2012 for five European countries—cinacalcet treatment resulted in increased lifetime cost of between € 23,878 to € 34,630 (22). Nevertheless, formal cost-effective analyses investigating efficacy and healthcare resource utilisation should be performed before conclusive assertions can be made about the economic advantages of PEIT and PCIT over surgical parathyroidectomy or life-long medical treatment.

In terms of efficacy in the four patients who underwent PEIT, one patient (25%) had successful ethanol ablation of his parathyroid adenoma after a single course, with no need for further treatment. Two patients (50%) had partial biochemical response, but required continued cinacalcet treatment. PEIT for the remaining patient (25%) was unsuccessful. These results are comparable to that in literature—Tanaka et al. reported in a 2008 retrospective cohort study of 104 SHPT patients undergoing PEIT that 30% were successfully managed with normalisation of serum calcium, phosphate and iPTH levels, 48% had biochemical improvement with PEIT but without complete normalisation, while 22% required parathyroidectomy (9).

Older reports of PEIT suggest a success rate of 42-50% when defined as a >30% reduction in iPTH level (23, 24). Using this benchmark, three out of five PCIT patients (60%) in our series had successful procedure, although one subsequently developed refractory disease. PCIT could not be repeated for the elderly patient who died shortly after the initial treatment from pneumonia, while PCIT allowed for improved HPT control and a lower cinacalcet dose in another patient. Nakanishi et al. reported PCIT in a series of nine patients with SHPT—two-thirds had improvement of iPTH to <38.2 pmol/L, while one-third was unsuccessful. In this study, the calcitriol used was of the same concentration as our series, but at a higher dose of 200-300% of calculated parathyroid gland volume and with at least three courses completed each within four weeks of the other (12).

In patients who responded, peak decrease in serum iPTH and calcium occurred at 1 month post-procedure. While effects of PEIT appear to be more sustained, especially in PHPT, serum iPTH and calcium trended upwards after 3 to 6 months in PCIT patients. This suggests the role for a repeat dose of PCIT after a time interval of 3 to 6 months for sustained efficacy, which would likely be acceptable to patients given the safer risk profile of PCIT compared to PEIT or surgery, and ambulatory nature of the procedure.

Both PEIT and PCIT are associated with risks of pain, bleeding and haematoma formation (9, 10, 25). The main concerns with PEIT are that of RLN injury and subsequent difficulty in the event of future parathyroidectomy due to ethanol-induced tissue fibrosis (9, 10, 12, 13, 15, 18). On the contrary, the few available studies on PCIT have not demonstrated risk of RLN injury, and this was consistent with our series (9–14). This is likely explained by the mechanism by which injected ethanol and calcitriol exercise their effects—PEIT causes coagulative necrosis and fibrous organisation within the injected parathyroid gland, and uncommonly capsular fibrosis with adherence to surrounding tissue including the RLN (12, 26). On the contrary, PCIT is postulated to directly increase calcitriol concentration within injected parathyroid glands, which causes parathyroid cell apoptosis and overcomes calcitriol-resistance with resultant decrease in synthesis and secretion of PTH (10, 12, 16).

The main limitations of our series are the small sample size, and heterogeneity in HPT characteristics, number and volume of anomalous parathyroid glands, baseline disease demographics including type and duration of dialysis for renal HPT patients, and pre-operative biochemistries which would reflect degree of metabolic bone disease. This Case Series report intends to evaluate PCIT and PEIT in the management of HPT in our specific institution’s experience in an ambulatory setting. However, these treatment modalities only serve as an alternative to surgery for patients who are not fit for General Anaesthesia or patients who decline surgical management. As such, this has led to a reduction in sample size and heterogeneity. The reduced follow-up duration of the study was attributed to the patient selection for this procedure, which preferentially results in a cohort of participants who have reduced life expectancy with poorer medical co-morbidities and compliance to follow up. In addition, the heterogeneity of our patient data and small sample size precludes utilisation of statistical analysis. Further well-designed clinical trials need to be performed to evaluate the efficacy and safety of PCIT in patients with renal HPT and PHPT prior to widespread adoption of this technique, including timing of repeat dosing for PCIT. For now, the role of percutaneous injection therapy appears confined to medication-resistant HPT patients with prohibitive surgical risk, or with a short life-expectancy. Dedicated studies could compare the cost-effectiveness of percutaneous injection therapy with surgical parathyroidectomy and life-long medical therapy. There have also been discussions for Radiofrequency ablation as a possible alternative treatment for HPT, but this has been excluded from our study as it is an invasive procedure which carries risks of recurrent laryngeal nerve injury and skin burns and as such, it is currently not practiced in our institution.

## Conclusion

PEIT and PCIT are feasible and safe therapeutic alternatives to surgical parathyroidectomy in HPT refractory to medical treatment, with postulated benefits of decreased costs and being an ambulatory procedure, without the requirement of hospitalisation or surgery under general anaesthesia. However, these findings are preliminary and require further future studies for more conclusive substantiation of the feasibility of PEIT/PCIT as non-surgical alternative treatment options. Cautious interpretation is required as this is pending large-scale prospective validation and further clinical trials need to be performed to evaluate efficacy and cost-effectiveness in these techniques prior to widespread adoption.

## Data Availability

Requests to access the datasets should be directed to eugene.leong@mohh.com.sg.
